# 17α-Methyltestosterone Affected Growth, Gonadal Development, and Intestinal Microbial Analysis in the Giant Freshwater Prawn (*Macrobrachium rosenbergii*)

**DOI:** 10.3390/ani15060870

**Published:** 2025-03-18

**Authors:** Bai Liufu, Qiyao Su, Kunhao Hong, Jie Wei, Yakun Wang, Zhiqiang Han, Lingyun Yu

**Affiliations:** 1 School of Fishery, Zhejiang Ocean University, Zhoushan 316000, China; cypressliufu@foxmail.com (B.L.); hanzq@zjou.edu.cn (Z.H.); 2 Key Laboratory of Tropical and Subtropical Fishery Resources Application and Cultivation, Ministry of Agriculture and Rural Affairs, Pearl River Fisheries Research Institute, Chinese Academy of Fishery Sciences, Guangzhou 510380, China; 15277124729@163.com (Q.S.); m15775090244@163.com (K.H.); weijie929ezio@outlook.com (J.W.); wykzkyky@163.com (Y.W.)

**Keywords:** 17α-methyltestosterone, *Macrobrachium rosenbergii*, gonadal development, histological analysis, sex ratio, intestinal flora

## Abstract

17α-methyltestosterone (MT) is known for its ability to suppress ovaries and induce spermatogenesis; yet, its effects in crustaceans are underexplored. This study investigates the impact of varying MT concentrations on the gonadal development and intestinal microbiota of juvenile *Macrobrachium rosenbergii*. Feeds containing different MT doses were provided, and the sex ratios, histological observations, reproductive gene expression, and intestinal microbial composition were analyzed. The results revealed short-term feeding (60 days) of 1000 mg/kg MT resulted in the highest male ratio, while long-term feeding (150 days) of 500 mg/kg MT achieved the same outcome. Conversely, long-term feeding of 1500 mg/kg MT led to the lowest male ratio and retarded male germ cell development. An intestinal microbiota analysis showed that MT supplementation significantly increased microbial abundance and altered the intestinal microbial community structure. Additionally, MT suppressed the expression of female reproductive-related genes. This study provides insights into the effects of MT on reproductive development and gut microbiota in juvenile prawns, offering a valuable reference for the application of MT in crustacean aquaculture.

## Simple Summary

17α-methyltestosterone (MT) is known to suppress ovaries and induce spermatogenesis; yet, its effects in crustaceans remain underexplored. This study investigates the impact of dietary MT on gonad development and intestinal microbiota in juvenile *Macrobrachium rosenbergii* through sex ratio determination, histological observation, the expression of reproductive development-related genes, and an analysis of the intestinal microbiota structure. The results showed that an. optimal MT concentration and treatment duration promoted gonadal differentiation and growth. However, excessive MT concentrations inhibited male gonad development and slowed growth rates. MT supplementation significantly increased microbial abundance and altered the intestinal microbial community structure. These findings clarify the effects of adding MT to feed on the reproductive development and intestinal microbiota of juvenile *M. rosenbergii*, providing a reference for MT application in crustaceans.

## 1. Introduction

Steroid hormones are lipophilic, small-molecule compounds secreted by endocrine glands into the bloodstream. They act as trace chemical messengers on specific target organs, including the hypothalamus, pituitary gland, adrenal glands, gonads, thyroid gland, parathyroid gland, and pancreas [1,2,3]. Based on the receptors they bind to, steroid hormones are classified into two main categories: sex hormones (including estrogens, androgens, and progestogens) and corticosteroids (including glucocorticoids and mineralocorticoids) [4,5]. Among these, sex hormones play a critical role in maintaining the reproductive system, while corticosteroids regulate carbohydrate and lipid metabolism, maintain the water–salt balance, and mediate stress responses [6,7]. Steroid hormones can be categorized based on their origin: endogenous hormones, which are naturally synthesized by the body to maintain homeostasis, and exogenous hormones, which are either synthetically produced or derived from other organisms to regulate biological metabolism, growth, and development [8,9,10]. Overall, steroid hormones play a crucial role in regulating growth, reproductive development, metabolism, and immune function.

Current research on steroid hormones has established a solid foundation for studying sex hormones, mainly concentrating on the following four aspects [11,12,13,14,15]. First, in the context of growth and development, the administration of estrogen (diethylstilbestrol) has been shown to accelerate growth and increase body weight in female *Monopterus albus*, while also enhancing egg production [16]. Second, in terms of gene expression related to reproductive development, feeding with androgen (methyltestosterone) reduces the expression levels of reproductive-development-related genes such as *Cyp19a1a*, *Foxl2* and *Foxl3* in female *Siniperca chuatsi*, while feeding with estrogen (estradiol) upregulates genes such as *Dmrt1*, *Sox9*, and *Amh* in *Astyanax altiparanae* [17,18]. Third, in the analysis of intestinal microbial composition, the injection of androgen (testosterone propionate) reduces intestinal microbiota diversity in female mice, significantly increasing *Firmicute* levels [19]. Fourth, in sex control, androgen (methyltestosterone) feeding induces sex reversal in species such as *Oreochromis niloticus* and results in monosex breeding [20]. However, research on sex hormones in crustaceans is limited. For instance, Jin et al. found that feeding *Macrobrachium nipponense* with estrogen (estradiol) and androgen (methyltestosterone) significantly increased growth rates, while high concentrations of sex hormones hindered normal testicular and ovarian development [21]. Similarly, Cai et al. reported that feeding with androgen (methyltestosterone) led to the presence of spermatocytes in the female gonads of *M. nipponense*, and the expression of genes such as *Dmrt11E*, *Foxl2*, and *SoxE1* increased [22]. In conclusion, while research on sex hormones in areas such as animal reproductive development, intestinal microbiota, and sex control is significant, studies on crustaceans remain scarce.

The giant freshwater prawn (*Macrobrachium rosenbergii*; *Crustacea*; *Decapoda*; *Palaemonidae*) is an important species for freshwater aquaculture in China. According to the 2023 China Fishery Statistical Yearbook, the production of *M. rosenbergii* in China exceeded 190,000 tons, ranking fourth in production among freshwater aquaculture Crustacean species. It has become one of China’s most economically important aquatic products, contributing to a significant consumer market [23]. *M. rosenbergii* exhibits sexual dimorphism, with sexually mature male prawn weighing approximately twice as much as females. Consequently, male prawn breeding generates significantly greater economic benefits than female prawn breeding [24,25]. Therefore, research into sex control technology to achieve monosex culture is of great practical value.

Studies on sex control techniques for *M. rosenbergii* have been reported. For instance, in an all-male culture, Sagi et al. demonstrated that the sex of male *M. rosenbergii* can be reversed to female by removing the androgenic gland, yielding an all-male group after hybridization. Subsequently, Ventura et al., successfully reversed the sex of male *M. rosenbergii* into pseudo-female individuals by injecting *Mr-IAG dsRNA*, resulting in an all-male group after hybridization [26,27]. In all-female culture, Levy et al. injected a cell suspension of the androgenic gland into juvenile prawns to obtain pseudo-male individuals, which were then used to generate all-female populations after hybridization [28]. These methods enable precise sex-reversed individuals, but involve complex procedures, such as the removal and transplantation of the androgenic gland, or the injection of dsRNA or cell suspension.

However, research on obtaining a monosex *M. rosenbergii* culture through the oral administration of sex hormones is limited, with only three related reports. First, feeding diets containing methyltestosterone increased the proportion of males [29]. Second, Baghel et al. found that feeding *M. rosenbergii* with diets containing various concentrations of methyltestosterone significantly increased the proportion of males [30]. Third, Ohs et al. found no significant difference in the male-to-female ratio after feeding *M. rosenbergii* a diet containing methyltestosterone [31].

Therefore, the objective of this study was to explore the effects of the androgen methyltestosterone on the reproductive development, sex ratio, sex-related gene expression, and intestinal microbial composition in *M. rosenbergii*, in order to provide insights for the application of sex hormones in crustaceans.

## 2. Materials and Methods

### 2.1. Dietary Preparation

The prawns were fed a commercial diet (Xiamen Hailin Biotechnology Co., Hailin, Xiamen, China) composed primarily of fish meal, soybean meal, peanut meal, flour, fish oil, soybean oil, and soybean lecithin. The 17α-methyltestosterone (MT, CAS No.: 58-18-4, purity: ≥97%) was obtained from Shanghai Aladdin Bio-Chem Technology Co., Ltd. (Shanghai, China). MT was added to each kilogram of diet at concentrations of 500 mg, 1000 mg and 1500 mg in groups E1, E2, and E3, respectively, with three replicates per group. The required amount of MT was weighed and dissolved in anhydrous ethanol, using 10% of the diet weight (i.e., 1 mL of ethanol per 10 g of diet). The diet was then immersed in the ethanol-MT solution and placed in a fume hood, away from light. After approximately 15 min, when most of the ethanol had evaporated, the feed was transferred to a tray, spread into a thin layer, and kept in the fume hood to complete the evaporation of the remaining ethanol in the dark. Finally, the diet was stored in a plastic container at 0 °C. The control group diet was prepared in the same manner, except that no MT was added to the ethanol before soaking and evaporation [31].

### 2.2. Experimental Animals and Design

Juvenile giant river prawn (weighing 9.30–9.40 mg) were obtained from the Pearl River Fisheries Research Institute. The rearing density was 500 individuals per group, and the rearing period lasted 150 days. The freshwater rearing system consisted of 12 tanks (130 cm × 50 cm × 60 cm), with aerated tap water as the experimental water. The water temperature was maintained at 29 ± 1 °C. The initial feeding rate was 30% of the total weight, gradually reduced to 5% as the prawn grew. The feed was provided twice daily (9 a.m. and 5 p.m.) [31]. Water quality parameters such as pH, ammonia nitrogen, nitrite, dissolved oxygen, and total alkalinity were analyzed using an Octadem W-II water quality analyzer (Octadem Technology, Inc., Wuxi, China) along with its dedicated reagents. The feeding behavior of the juvenile prawns was observed in real time, and uneaten feed and feces were promptly removed using a siphon to maintain water quality.

### 2.3. Analysis of Reproductive-Related Gene Expression Levels

The experiment was conducted for 150 days. Gonad samples from 18 prawns (9 male and 9 female) were randomly collected from each group and stored in liquid nitrogen. Total RNA was extracted from gonad tissues using the Trizol Reagent kit (Invitrogen, Waltham, MA, USA). Tissues were homogenized in Trizol, followed by chloroform-mediated phase separation. RNA was precipitated with isopropanol, washed with 75% ethanol, and dissolved in RNase-free water. RNA purity and concentration were verified via spectrophotometry. For cDNA synthesis, 1 µg RNA was reverse-transcribed using the M-MLV reverse transcriptase kit (Invitrogen, Waltham, MA, USA). Reactions included oligo(dT) primer annealing (65 °C, 5 min), reverse-transcription (37 °C, 50 min), and enzyme inactivation (70 °C, 15 min). cDNA was stored at −20 °C. Table 1 lists the primer sequences for the gene related to reproductive development. The *β-actin* gene (GenBank accession number: AY651918.2) was used as the internal reference gene. The real-time fluorescence quantitative PCR assay system consisted of 1 μL of cDNA (50 ng/μL), 10 μL of iTaq Universal SYBR Green Supermix, 1 μL of primer (10 pmol/μL), and 7 μL of double steaming water. The qPCR reaction conditions were as follows: initial preheating at 95 °C for 3 min, denaturation at 95 °C for 40 s, annealing at 60 °C for 45 s, and extension at 72 °C for 30 s, followed by a final extension at 72 °C for 10 min. A total of 35 cycles were performed. Gene expression was normalized by calculating the Ct values for each reaction using the 2^−ΔΔCt^ method.

### 2.4. Measurement of the Growth Traits

On the 1st and 150th days of the experiment, body weights of 10 randomly selected prawns from each replicate were measured using a Mettler Toledo AL-204 precision balance (Mettler Toledo, Inc., Shanghai, China) (Table 2). The weight growth rate and specific growth rate of each group were calculated using the formula provided by Cai et al. [22].

The weight growth rate (WGR, %) was determined as follows:

WGR = (W_t_ − W_0_)/W_0_ × 100%

The specific growth rate (SGR, %/d) was determined as follows:

SGR = (In W_t_ − In W_0_)/t × 100%

where W_t_ is the average body weight (g) on the 150th day of the experiment, W_0_ is the average body weight (g) on the 1st day of the experiment, and t is the total days of culture.

### 2.5. Sex Ratio Statistics

On the 60th and 150th days, 30 prawns were randomly selected from each group to determine the sex ratio. The criteria for determining the sex of prawns followed those outlined by Shen et al. [32]. Male prawns were identified based on the following characteristics: the second pereiopods (claw) are longer, the genital pores are located on the fifth pereiopods, and a rod-shaped protruding accessory is present at the inner edge of the second pleopods. Female prawns were identified by the following characteristics: the genital pores are located on the third pereiopods, the distance between the fifth pereiopods is wider, and the female prawns lack male appendages.

### 2.6. Histological Observations of Gonadal Development

Gonad samples from 30 prawns per group were collected on the 150th day and fixed in Bouin’s fixative for 2 h. Subsequently, the samples underwent dehydration, xylene transparency, and paraffin embedding. Sections (7 μm) were prepared using a Leica RM2016 paraffin microtome (Leica, Weztlar, Germany). The sections were stained with hematoxylin–eosin (HE) solution (Nanjing Jiancheng Bioengineering Institute, Nanjing, China) and then sealed with neutral gum for preservation. Histological changes in the testes and ovaries were observed under an optical microscope (Nikon, Tokyo, Japan). Different cell types were compared and identified based on their cellular morphology.

### 2.7. Analysis of Intestinal Microbial Diversity

Full intestinal samples from 30 prawns per group were collected on the 150th day and stored in liquid nitrogen. Genomic DNA was extracted from the intestines following the operational procedures of the Omicsmart platform. The V3–V4 hypervariable region of 16S rDNA was amplified using specific primers with barcodes. The selected primer sequences were 341F: (CCTACGGGNGGCWGCAG) and 806R: (GGACTACHVGGGTATCTAAT). PCR amplification of the target region, MiSeq library construction, MiSeq sequencing, quality control, and basic operational taxonomic unit (OTU) analysis were performed. Based on OTU sequence and abundance data, species annotation, α-diversity analysis, and microbial difference analyses among groups were conducted at the phylum and genus levels.

### 2.8. Statistical Analysis

Experimental data are expressed as mean ± standard error of the mean (SEM), and results were analyzed using Graphpad Prism 9.5 (GraphPad Software Inc., San Diego, CA, USA). Group comparisons were analyzed using one-way ANOVA, supplemented with LSD and Duncan’s tests, with statistical significance set at *p* < 0.05.

## 3. Results

### 3.1. Effects of MT Concentration on the Sex Ratio of M. rosenbergii

The sex ratio (male:female) at different MT concentrations and time points is illustrated in Table 3. After 60 days of feeding, the sex ratios for the control group, E1 group, E2 group, and E3 group were 1.94 ± 0.17, 1.78 ± 0.20, 2.55 ± 0.16, and 2.05 ± 0.20, respectively. The sex ratio of E2 group was significantly higher than that of the control group (*p* < 0.05), with no significant differences observed between the other groups (*p* > 0.05). After 150 days of MT feeding, the sex ratios of the control, E1, E2, and E3 groups ranged from 0.93 to 2.66. The sex ratio of the E1 group was significantly higher than that of the control and E2 groups (*p* < 0.05), and significantly higher than that of the E3 group (*p* < 0.01).

### 3.2. Histological Observations of the Gonad

**Figure 1:**
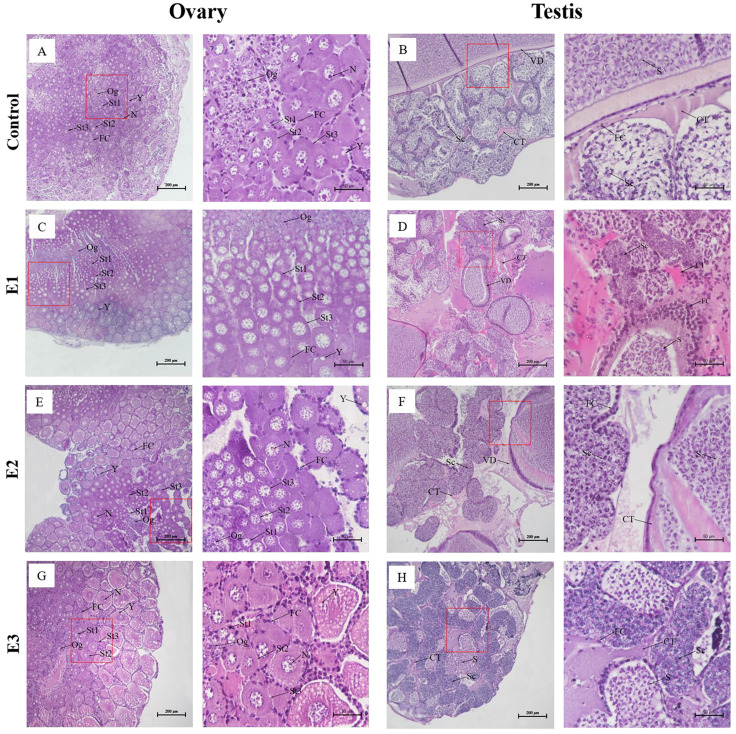
Histological sections of (**A**) female prawn ovary in the control; (**B**) male prawn testis in the control; (**C**) female prawn ovary in the 500 mg/kg group; (**D**) male prawn testis in the 500 mg/kg group; (**E**) female prawn ovary in the 1000 mg/kg group; (**F**) male prawn testis in the 1000 mg/kg group; (**G**) female prawn ovary in the 1500 mg/kg group; and (**H**) male prawn testis in the 1500 mg/kg group. (**A**–**H**) The right image shows high-magnification views of areas enclosed in red boxes in the images to the left. N: nucleus; Og, oogonia; St1, stage-1 oocytes; St2, stage-2 oocytes; St3, stage3 oocytes; Sc: spermatogonia; S: sperm; VD: vas deferens; FC: follicle cells; Y: yolk granule; CT: connective tissue. Scale bars: 200 μm and 50 μm.

Histological observation showed that the gonads of the control females contained stage I, II, and III oocytes, as well as mature oocytes, in which yolk granules appeared (Figure 1A). The gonads of female prawn fed the MT diet also contained stage I, II, and III oocytes, as well as mature oocytes. The morphology and number of oocytes at each stage were similar to those of the control group (Figure 1C,E,G). The male gonads of the control, E1, and E2 groups contained primary spermatocytes, secondary spermatocytes, and sperm (Figure 1B,D,F). The male gonads of the E3 group contained spermatogonia, primary spermatocytes, secondary spermatocytes, and sperm. Most of the germ cells in this group were at the spermatogonia stage of development (Figure 1H).

### 3.3. Effect of MT Concentration on the Growth Traits of M. rosenbergii

The overall growth of the four groups is presented in Table 2. The final body weights of the control, E1, E2, and E3 groups were 9.47 ± 1.20 g, 8.27 ± 0.66 g, 9.98 ± 0.78 g, and 11.07 ± 0.93 g, respectively. For the WGR and SGR, the values were 101,421.97 ± 12,840.77% and 4.48 ± 0.05%/d in the control group, 88,528.08 ± 7025.70% and 4.49 ± 0.05%/d in the low-dose E1 group, 106,481.61 ± 8277.65% and 4.66 ± 0.05%/d in the medium-dose E2 group, and 118,086.47 ± 9955.57% and 4.72 ± 0.06%/d in the high-dose E3 group. The high-dose E3 group showed significantly elevated WGR and SGR levels compared to the control and low-dose E1 groups (*p* < 0.05), but there were no significant differences between the high-dose E3 group and medium-dose E2 group (*p* > 0.05). The WGR and SGR of the medium-dose E2 group and the control were significantly higher than those of the low-dose E1 groups (*p* < 0.05), but there were no significant differences between the control and medium-dose E2 group (*p* > 0.05).

The effects of MT on the growth of male prawn are presented in Table 2. The final body weights of the control group, E1 group, E2 group, and E3 group were 13.55 ± 0.72 g, 9.90 ± 0.61 g, 9.94 ± 0.50 g, and 10.17 ± 1.13 g, respectively. For WGR, the values for the four groups ranged from 105,990.53% to 177,965.93 ± 18,392.46%. The SGR of the control, E1, E2, and E3 groups were 4.95 ± 0.07%/d, 4.64 ± 0.04%/d, 4.64 ± 0.03%/d, and 4.67 ± 0.07%/d, respectively. The growth rate and SGR of the three MT-fed groups were significantly lower than those of the control group (*p* < 0.05). The effects of MT on the growth of female prawns are presented in Table 2. The final body weight of the control, E1, E2, and E3 groups ranged from 6.76g to 11.59 g. The WGR and SGR of the four groups ranged from 72,286.51% to 123,676.73% and 4.23%/d to 4.77%/d, respectively. The WGR in the high-dose E3 group was significantly elevated relative to both the low-dose and control groups (*p* < 0.05). Furthermore, the SGR values in the E2 and E3 groups were significantly higher than those observed in the control and E1 groups (*p* < 0.05), while no significant difference in the SGR were observed among the three MT-fed groups (*p* > 0.05).

### 3.4. Effects of MT Concentration on Intestinal Microbial Diversity of M. rosenbergii

Table 4 presents the effects of MT feeding on the alpha diversity index of prawn. The Sobs and Simpson indices for the four groups (control group, E1 group, E2 group, and E3 group) ranged from 469.50 to 796.00 and from 0.85 to 0.91, respectively. The Sobs and Simpson index of the three MT-fed groups were significantly higher than those of the non-MT-fed group (*p* < 0.05). The Chao1 index for the four groups ranged from 526.51 to 952.00. The Chao1 index of the E3 group was significantly higher than that of the E1 and E2 groups (*p* < 0.05), and significantly higher than that of the control group (*p* < 0.01). The Shannon indices for the control group, E1 group, E2 group, and E3 group were 3.65 ± 0.04, 4.65 ± 0.05, 4.24 ± 0.18, and 4.82 ± 0.06, respectively. The Shannon index of E1 and E3 groups was significantly higher than that of the E2 group (*p* < 0.05), and significantly higher than that of control group (*p* < 0.01). The sequencing depth coverage index for the four groups ranged from 99.82% to 99.86%.

**Figure 2:**
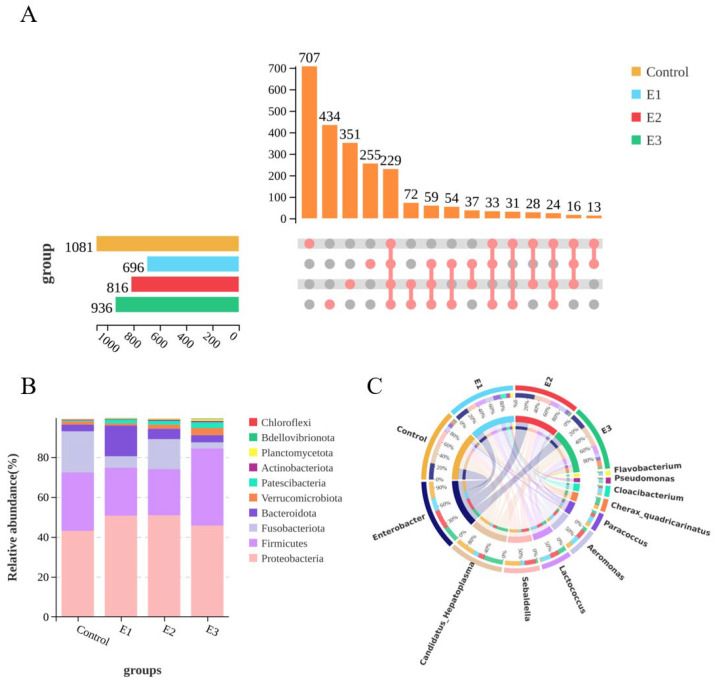
Effects of different concentrations of MT on intestinal microbiota of *M. rosenbergii* for 150 days. (**A**) UpSet plot comparing the relationships of intestinal microbiota OTUs at different MT concentrations. The left panel displays colorful bars for each treatment, representing the number of OTUs. Pink circles in the matrix indicate sets of treatments that intersect. (**B**) Taxonomic composition of gut bacterial communities in four groups at the top 10 phylum level. (**C**) Taxonomic composition of gut bacterial communities in four groups at the top 10 genus level.

A total of 3529 OTUs were generated from the control, E1, E2, and E3 groups, with 229 common OTUs annotated across the four groups. The control, E1, E2, and E3 groups had 707, 443, 351, and 255 unique OTUs, respectively (Figure 2A). A relative abundance analysis was conducted at the phylum level. The composition characteristics of the top ten phyla by abundance in the gut after MT feeding are shown in Figure 2B. The dominant phyla across all four groups were Proteobacteria, Firmicutes, Fusobacteriota, Bacteroidota, and Verrucomicrobiota. The level of Proteobacteria in E1 and E2 groups was significantly higher than that in the control group and E3 group (*p* < 0.05). The level of Firmicutes in the E3 group was significantly elevated compared to the control group (*p* < 0.05) and markedly higher than that in the E1 and E2 groups (*p* < 0.01). Conversely, the level of Fusobacteriota in the control group exceeded that in the E2 group (*p* < 0.05) and was significantly greater than in the E1 and E3 groups (*p* < 0.01). Additionally, the level of Bacteroides in the E1 group was significantly higher than in the other three groups (*p* < 0.05). The composition characteristics of the top ten bacterial genera in the gut after MT feeding are shown in Figure 2C. The common dominant genus across the four groups was *Enterobacter*, its abundance exceeding 18.04%. There were no significant differences among the groups (*p* > 0.05). Additionally, *Candidatus_Hepatoplasma* and *Sebaldella* were the specific dominant genera in the control group, while *Lactococcus* and *Aeromonas* were the specific dominant genera in the E1 group. The specific dominant genera in E2 group were *Sebaldella*, *Aeromonas*, and *Lactococcus*, while *Enterobacter* and *Lactococcus* were the specific dominant genera in the E3 group.

### 3.5. Gene Expression Levels Associated with Reproduction

**Figure 3:**
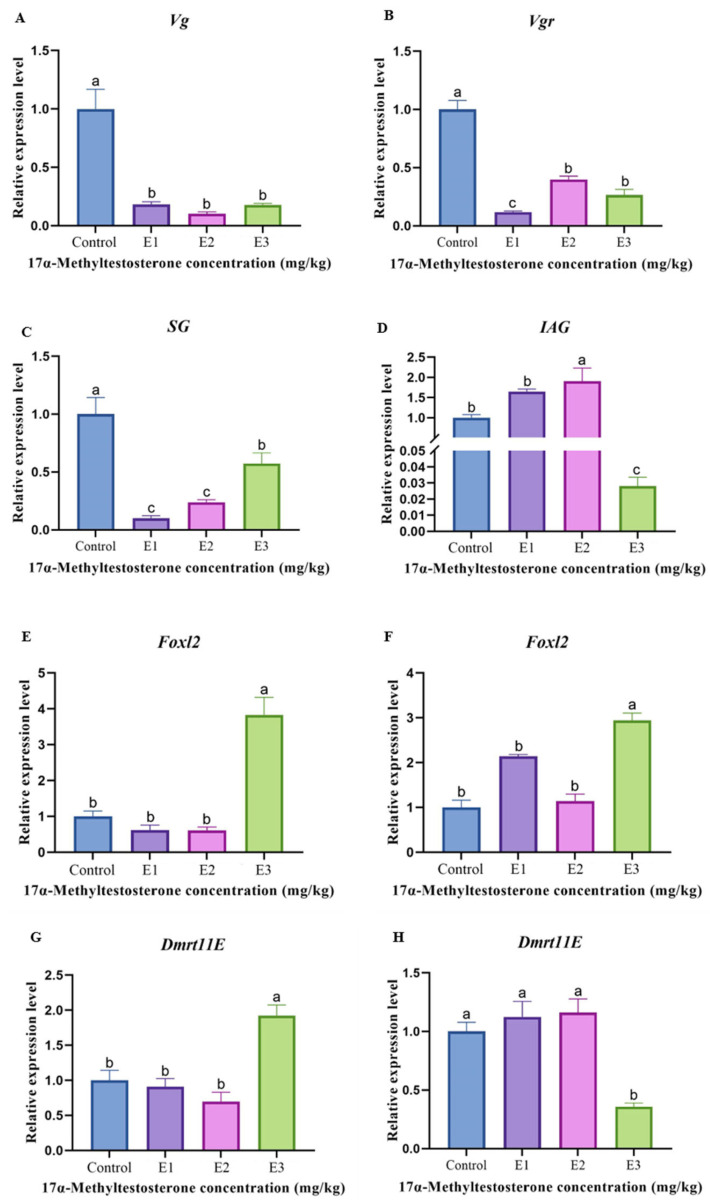
Expression characterization of different genes in female and male prawns treated with varying MT concentrations for 150 days. (**A**,**B**) *Vg* and *Vgr* expression levels in female prawns; (**C**,**D**) *SG* and *IAG* expression levels in male prawns; (**E**,**F**) *Foxl2* expression levels in female and male prawns; (**G**,**H**) *Dmrt11E* expression levels in female and male prawns. Data are presented as mean ± SEM from individual prawns per group (n = 3). The different letters indicated a significant difference between the experimental groups at the same time (*p* < 0.05).

The gene expression levels associated with the reproductive development of prawn after MT feeding are shown in Figure 3. Regarding female reproductive and developmental genes, the expression level of the *Vg* gene in the non-MT-fed group was significantly higher than that in the MT-fed group (*p* < 0.05) (Figure 3A). The expression level of the *Vgr* gene in the non-MT-fed group was significantly higher than that in the medium- and high-dose groups (*p* < 0.05), and significantly higher than that in the low-dose group (*p* < 0.01) (Figure 3B). Regarding male reproductive and developmental genes, the SG expression level in the non-MT-fed group was significantly higher than that in the high-dose group (*p* < 0.05), and significantly higher than that in the medium- and low-dose groups (*p* < 0.01) (Figure 3C). The expression level of the *IAG* gene in the middle-dose group was significantly higher than that in the non-MT-fed group and the low-dose group (*p* < 0.05), and was significantly higher than that in the high-dose group (*p* < 0.01) (Figure 3D). Regarding both female and male reproductive development genes, the expression levels of the *Foxl2* gene in the female and male gonads of the high-dose MT-fed group were significantly higher than those of the other groups (*p* < 0.05) (Figure 3E,F). The expression level of the *Dmrt11E* gene in the female gonads in the high-dose MT-fed group was significantly higher than that in the other groups (*p* < 0.05) (Figure 3G). The expression level of the *Dmrt11E* gene in the male gonads of the control, low-dose, and medium-dose groups was significantly higher than that in the high-dose group (*p* < 0.05) (Figure 3H).

## 4. Discussion

17α-Methyltestosterone is capable of promoting and maintaining the development of male reproductive organs, as well as inducing spermatogenesis and sperm release. It is frequently used as a sexual inducer to masculinize female fish in the aquaculture industry [33,34,35]. During sex reversal, different aquatic species exhibit variations in their abilities to absorb and utilize MT. For example, a 10 mg/kg MT dose can successfully induce sex reversal in juvenile *Paralichthys olivaceus*, and the same concentration can also masculinize 4-month-old *Dicentrarchus labrox* [36,37]; *Oreochromis mossambicus* can achieve complete sex reversal at 30 mg/kg MT, though a short feeding duration can reduce the male ratio [38,39]. The optimal MT concentration for *Oreochromis niloticus* is 60 mg/kg, while, for *Poecilia reticulata*, *P. latipinna*, and other species in the *Poeciliidae* family, it is 90 mg/kg [40]. In crustacean research, feeding *M. nipponense* with 200 mg/kg MT consistently maintained a higher sex ratio (2.61:1), while feeding 400 mg/kg MT to *M. rosenbergii* led to a slight increase in the male proportion without significantly altering the sex ratio [22,31]. Thus, selecting the appropriate hormone concentration is crucial for the successful induction of sex reversal.

In this study, after 60 days of feeding, the highest male rate (71.83%) was observed at a concentration of 1000 mg/kg MT, making it the most suitable dose for short-term sex reversal. In contrast, after long-term feeding (150 days), the highest male rate (72.68%) occurred at a concentration of 500 mg/kg MT, which was the optimal dose for long-term sex reversal. Furthermore, this study found that, with prolonged feeding, the sex ratio of prawns fed with 1000 mg/kg and 1500 mg/kg MT showed a decreasing trend. The sex ratio in the 1500 mg/kg MT group was lower than that in the control group. Some studies suggest that long-term feeding of high concentrations of MT to aquatic animals can lead to the aromatization of methyltestosterone, resulting in the production of estrogen and, ultimately, a decline in the male ratio, as observed in species such as *Esox masquinongy*, *Pimephales promelas*, and zebrafish (*Danio rerio*) [41,42,43]. The results of this study are consistent with these findings. Thus, this study demonstrates that the optimal MT concentrations for achieving the highest male rate in *M. rosenbergii* are 1000 mg/kg (for short-term feeding) and 500 mg/kg (for long-term feeding).

Feeding MT can influence gonadal development and growth performance in aquatic animals, with varying effects depending on the dosage. For instance, administering low doses of MT to *Oreochromis spilurus* and *Epinephelus coioides* can stimulate their growth and accelerate testicular maturation [44,45,46]. In contrast, high doses of MT given to *P. promelas* results in developmental delays and testicular atrophy [47]. Research on *Strongylocentrotus intermedius* has shown that abnormal gonadal development can inhibit growth performance by influencing nutrient allocation and metabolism [48]. In the study of *M. nipponense*, MT was found to inhibit ovarian development and reallocate energy originally allocated to gonadal development toward growth, thereby enhancing growth in female prawns. In contrast, MT induced an over-reliance on exogenous hormones in male prawns, consequently resulting in delayed testicular maturation and reduced sperm production, and this substantial metabolic expenditure on gonadal development ultimately inhibited growth [22]. In this study, no abnormalities were observed in the male gonads of the low-dose and medium-dose groups. However, in the high-dose group, most male germ cells remained in the spermatogonia stage. Additionally, male prawn growth was delayed after feeding all three doses of MT. Regarding female gonad development, there were no differences in the gonad stages, cell morphology, or oocyte quantity of female prawns between the three MT-fed groups and the non-MT-fed group (Figure 1). No significant difference in growth was observed between the low-dose group and the non-MT-fed group (*p* > 0.05), while the body weights of female prawns in the medium-dose and high-dose groups were significantly higher (*p* < 0.05). The results of this study indicate that feeding MT to *M. rosenbergii* reduces the growth performance of male prawns, and feeding 1500 mg/kg MT also delays testicular development. However, while feeding 1000 mg/kg and 1500 mg/kg MT had no significant impact on the female gonads, it enhances the growth performance of female prawns. Therefore, we posit that the growth difference between male and female prawns is attributable to abnormal gonadal development, which influences nutrient allocation and metabolism.

The diversity and structure of the intestinal microbiota in aquatic animals are influenced by factors such as species, diet, and the external environment [49,50]. Due to the discharge of sewage and aquaculture effluents, coupled with the potential risk of steroid contamination arising from rapid population growth, sex hormones have been identified as key factors influencing intestinal microbiota [51,52]. For example, when zebrafish were exposed to estradiol, their intestinal microbiota diversity increased, with a significant rise in the abundance of the *CKC4* genus and a notable decrease in the abundance of the *Bacteroides* genus [53]. In this study, the alpha diversity indices (the Sobs, Shannon, Simpson, and Chao1 indices) of the intestinal microbiota of prawns fed MT significantly increased in all three groups (*p* < 0.05) (Table 4). The high-dose group (1500 mg/kg) exhibited a significantly higher relative abundance of Firmicutes compared with other groups, while the low-dose group (500 mg/kg) uniquely harbored Bacteroidota as the predominant phylum at the taxonomic level. These results suggest that the intestinal microbiota structure and abundance in *M. rosenbergii* were influenced by the sex hormone methyltestosterone. This study also found that the dominant phyla of prawn were *Proteobacteria* and *Firmicutes*, with *Proteobacteria* being the most predominant phylum. It has been reported that *Proteobacteria* play a crucial role in the process of intestinal immune function. For example, it was found that *Proteobacteria* contains potentially pathogenic bacteria, such as *Vibrionaceae* and *Rhodobacteraceae*, whose increased abundance would heighten the pathogenic risk in cultured organisms like *Litopenaeus vannamei*. However, the moderate stimulation of *Proteobacteria* can also enhance shrimp immunity [54]. The results of this study showed an increase in the abundance of *Proteobacteria* in the low-dose group and medium-dose groups, suggesting that adding low and medium doses of MT to the feed could increase the disease risk in *M. rosenbergii*. *Firmicutes* facilitates nutrient utilization in the intestine. For example, studies have shown that *Firmicutes* enhance nutrient absorption by increasing the number of epithelial lipid droplets in zebrafish [55]. In nutrient metabolism and absorption, *Firmicutes* can form a symbiotic relationship with *Bacteroidetes*. A high *Firmicutes*: *Bacteroidetes* (F:B) ratio can enhance intestinal lipid metabolism and energy absorption, and increase the fatty acid content of animals [56]. In this experiment, the F:B ratio of prawns fed with a high dose of MT was the highest (10.80), while the lowest was observed in the low-dose group (1.56). Therefore, it is hypothesized that prawns fed with a high dose of MT (1500 mg/kg) have a greater nutrient absorption capacity compared to those fed with a low dose (500 mg/kg).

Gonad development and sex determination is a complex process. Under natural conditions, variations in the sex ratio and gonadal development often limit effective resource management and progress in breeding programs. Steroid hormones can influence the gonadal development of aquatic organisms and may even induce sexual reversal [57]. In adult scallops (*Patinopecten yessoensis*), the *Foxl2* and *Tesk* genes are expressed in the gonads of mature males and females, respectively. Studies have shown that treatment with testosterone (T) and *17β*-estradiol (E2) can influence the *Tesk* expression in the gonads, but has no effect on other aspects of gonadal development. This suggests that sex hormone treatments may influence gonadal development during sexual reversal [58]. In this study, the long-term feeding of MT to *M. rosenbergii* altered the expression levels of six reproductive development genes, indicating that methyltestosterone can influence gonadal development in prawns. The expression levels of the *Vg* and *Vgr* genes in female reproductive development were significantly lower in MT-fed prawns compared to non-MT-fed prawns (*p* < 0.05), suggesting that methyltestosterone may inhibit the development of female gonads in *M. rosenbergii*. The expression levels of the *SG* and *IAG* gene in male reproductive development varied after MT feeding, indicating that methyltestosterone affects male gonads in prawns, with different doses having distinct effects. The gonadal development gene *Foxl2* was significantly highly expressed in both female and male gonads of prawns fed high-dose MT (*p* < 0.05). Additionally, the expression of the *Dmrt11E* gene was significantly higher in the male gonads of prawns fed high-dose MT, whereas the expression of *Dmtr11E* was significantly lower in the male gonads of prawns fed high-dose MT (*p* < 0.05). Considering the sex ratio (0.93 male-to-female ratio of gonads at high dose) (Table 3) and the histological results showing the stagnation of male germ cell development (Figure 1H), it is speculated that feeding high-dose MT to prawns is not beneficial for male gonad development or male ratio improvement. However, it appears to benefit the female ratio of *M. rosenbergii*. The molecular regulatory mechanisms underlying this effect require further investigation.

## 5. Conclusions

MT plays a crucial role in promoting the development of male reproductive organs, spermatogenesis, and sperm release in aquatic organisms. In this study, different MT concentrations were tested during the rearing of juvenile prawn, revealing varying effects on gonadal development. Specifically, long-term (150 days) feeding with 500 mg/kg MT and short-term (60 days) feeding with 1000 mg/kg were both effective in promoting male gonad differentiation and development. However, an excessively high dose of 1500 mg/kg MT inhibited male gonad development and slowed growth rates. Furthermore, feeding MT-containing diets suppressed the expression of female reproductive genes and significantly altered the intestinal microbiota diversity and structure in prawns. These findings suggest that MT can be a valuable tool for sex control and gonadal development in crustaceans, although the optimal dosing and feeding duration must be carefully considered to avoid negative effects on the gonadal and growth outcomes.

## Figures and Tables

**Table 1:** Primers used for qPCR in this study.

Primer Name	Sequence (5′→3′)	Sources	Product Size (bp)	Amplification Efficiency (%)	R^2^
*β-actin*-F	CAGGGAAAAGATGACCCAGA	AY651918.2	171	98.5	0.997
*β-actin*-R	GGAAGTGCATACCCCTCGTA				
*Vg*-F	GGACGCTGATCGTAACCC	AB056458.1	192	98.7	0.996
*Vg*-R	TACCTCTAGCATCAAACT				
*Vgr*-F	TACCTTAGCATCAAACT	GU454802.1	145	96.8	0.987
*Vgr*-R	GAGAAGGCGGTAAGTCTGGTT				
*Foxl2*-F	AGTCCCGACAGAAAGCTTCA	MZ647492.1	191	97.7	0.995
*Foxl2*-R	TGCCCAAAGATCCTCCGATT				
*IAG*-F	GGACAGCGTGAGGAGAAGTC	FJ409645.1	185	97.5	0.991
*IAG*-R	ACTAAAGCAGCGGGAAGACA				
*Dmrt11E-F*	ACCACCAGTAGCACCACAATACAACAG	KC801044.1	189	96.7	0.991
*Dmrt11E-R*	CAAGAATCTGAAGGAAGTTCGTGAGTG				
*SG*-F	CCACCCATTCCTGGTAAGCATCA	EF647641.1	104	99.2	0.998
*SG*-R	GAGTGTCCATTCGGTAACTCGTAG				

Note: *Vg*, Vitellogenin; *Vgr*, Vitellogenin receptor; *Foxl2*, Forkhead box L2; *IAG*, Insulin-like androgenic gland hormone; *SG*, Sperm gelatinase; *Dmrt11E*, Doublesex and mab-3 related transcription factor 11E.

**Table 2:** Effects of different concentrations of 17α-MT on growth performance of *M. rosenbergii*.

Index		Group			
		Control	E1	E2	E3
all	IMW (mg)	9.33 ± 0.78	9.30 ± 0. 97	9.37 ± 0.71	9.40 ± 0.60
	FMW (g)	9.47 ± 1.20 ^b^	8.27 ± 0.66 ^c^	9.98 ± 0.78 ^ab^	11.07 ± 0.93 ^a^
	WGR (%)	101,421.97 ± 12,840.77 ^b^	88,528.08 ± 7025.70 ^c^	106,481.61 ± 8277.65 ^ab^	118,086.47 ± 9955.57 ^a^
	SGR (%/d)	4.48 ± 0.05 ^b^	4.49 ± 0.05 ^b^	4.66 ± 0.05 ^ab^	4.72 ± 0.06 ^a^
male	FMW (g)	13.55 ± 0.72 ^a^	9.90 ± 0.61 ^b^	9.94 ± 0.50 ^b^	10.17 ± 1.13 ^b^
	WGR (%)	177,965.93 ± 18,392.46 ^a^	105,955.73 ± 6516.02 ^b^	105,990.53 ± 5360.25 ^b^	108,503.15 ± 10,113.37 ^b^
	GR (%/d)	4.95 ± 0.07 ^a^	4.64 ± 0.04 ^b^	4.64 ± 0.03 ^b^	4.67 ± 0.07 ^b^
female	MW (g)	6.76 ± 1.33 ^b^	7.19 ± 0.90 ^b^	10.01 ± 1.27 ^ab^	11.59 ± 1.33 ^a^
	WGR (%)	72,286.51 ± 14,271.60 ^b^	76,909.65 ± 9664.70 ^b^	106,809.00 ± 13,602.91 ^ab^	123,676.73 ± 14,240.77 ^a^
	SGR (%/d)	4.23 ± 0.14 ^b^	4.38 ± 0.07 ^ab^	4.65 ± 0.08 ^a^	4.77 ± 0.09 ^a^

Note: The data are expressed as mean ± SEM (n = 30). The different letters indicated a significant difference between the experimental groups at the same time (*p* < 0.05). IMW: initial mean weight. FMW: final mean weight. WGR: weight growth rate. SGR: specific growth rate.

**Table 3:** Male-to-female ratio of *M. rosenbergii* fed diets containing different concentrations of 17α-MT for different durations.

17α-Methyltestosterone Treatment Time (Days)	Group (mg/kg)			
	Control	E1	E2	E3
60	1.94 ± 0.17 ^b^	1.78 ± 0.20 ^b^	2.55 ± 0.16 ^a^	2.05 ± 0.20 ^b^
150	1.70 ± 0.55 ^b^	2.66 ± 0.29 ^a^	1.90 ± 0.21 ^b^	0.93 ± 0.06 ^c^

Note: Data are shown as mean ± SEM of tissues from separate individuals (n = 30). The different letters indicated a significant difference between the experimental groups at the same time (*p* < 0.05).

**Table 4:** Effects of different concentrations of 17α-MT on the diversity index of *M. rosenbergii* for 150 days.

Index	Group			
	Control	E1	E2	E3
Sobs	469.50 ± 29.50 ^b^	615.00 ± 38.18 ^a^	700.33 ± 25.83 ^a^	796.00 ± 122.53 ^a^
Shannon	3.65 ± 0.04 ^c^	4.65 ± 0.05 ^a^	4.24 ± 0.18 ^b^	4.82 ± 0.06 ^a^
Simpson	0.85 ± 0.00 ^b^	0.91 ± 0.01 ^a^	0.89 ± 0.03 ^a^	0.90 ± 0.00 ^a^
Chao1	526.51 ± 23.41 ^c^	630.95 ± 36.64 ^b^	755.98 ± 25.52 ^b^	952.00 ± 19.28 ^a^
Coverage (%)	99.86 ± 0.00	99.83 ± 0.01	99.82 ± 0.00	99.84 ± 0.00

Note: The different letters indicated a significant difference between the experimental groups at the same time (*p* < 0.05).

## Data Availability

The data presented in this study are available upon request from the corresponding author.
